# Combination of a Collagen Scaffold and an Adhesive Hyaluronan-Based Hydrogel for Cartilage Regeneration: A Proof of Concept in an Ovine Model

**DOI:** 10.1177/1947603521989417

**Published:** 2021-01-29

**Authors:** Clara Levinson, Emma Cavalli, Brigitte von Rechenberg, Marcy Zenobi-Wong, Salim E. Darwiche

**Affiliations:** 1 Tissue Engineering and Biofabrication, Institute for Biomechanics, Swiss Federal Institute of Technology Zurich (ETH Zurich), Zurich, Switzerland; 2 Musculoskeletal Research Unit (MSRU), Vetsuisse Faculty, University of Zurich, Zurich, Switzerland; 3 Center for Applied Biotechnology and Molecular Medicine (CABMM), University of Zurich, Zurich, Switzerland

**Keywords:** hyaluronan, collagen, chondral defect, in situ regeneration, ovine study

## Abstract

**Objective:**

Hyaluronic acid–transglutaminase (HA-TG) is an enzymatically crosslinkable adhesive hydrogel with chondrogenic properties demonstrated *in vitro* and in an ectopic mouse model. In this study, we investigated the feasibility of using HA-TG in a collagen scaffold to treat chondral lesions in an ovine model, to evaluate cartilage regeneration in a mechanically and biologically challenging joint environment, and the influence of the surgical procedure on the repair process.

**Design:**

Chondral defects of 6-mm diameter were created in the stifle joint of skeletally mature sheep. In a 3-month study, 6 defects were treated with HA-TG in a collagen scaffold to test the stability and biocompatibility of the defect filling. In a 6-month study, 6 sheep had 12 defects treated with HA-TG and collagen and 2 sheep had 4 untreated defects. Histologically observed quality of repair tissue and adjacent cartilage was semiquantitatively assessed.

**Results:**

HA-TG adhered to the native tissue and did not cause any detectable negative reaction in the surrounding tissue. HA-TG in a collagen scaffold supported infiltration and chondrogenic differentiation of mesenchymal cells, which migrated from the subchondral bone through the calcified cartilage layer. Additionally, HA-TG and collagen treatment led to better adjacent cartilage preservation compared with empty defects (*P* < 0.05).

**Conclusions:**

This study demonstrates that the adhesive HA-TG hydrogel in a collagen scaffold shows good biocompatibility, supports *in situ* cartilage regeneration and preserves the surrounding cartilage. This proof-of-concept study shows the potential of this approach, which should be further considered in the treatment of cartilage lesions using a single-step procedure.

## Introduction

Articular cartilage has a limited ability to self-repair after injuries. This is partly due to its avascular nature that prevents progenitor cells in blood to migrate to the site of the lesion and the limited number of resident cells.^1^ If left untreated, cartilage lesions often lead to joint degeneration and eventually to the development of posttraumatic osteoarthritis.^2^ About 60% of patients undergoing knee arthroscopic surgery and up to 69% of adults older than 50 years show signs of cartilage anomalies in their knees.^3^ The limited capability of cartilage to heal has driven the development of tissue engineering strategies such as microfracture, autologous chondrocyte implantation (ACI), and matrix-assisted autologous chondrocyte implantation (MACI).^4,5^ However, these techniques are often unavailable due to their high costs and their long-term outcome is variable or unknown.^6^ Furthermore, they present major limitations, which include formation of mechanically inferior tissue like fibrocartilage,^6,7^ lack of integration of the grafts, the requirement of multiple surgeries and high donor-to-donor variability.^8^ Cell-free approaches, using smart biomaterials able to recruit chondrogenic cells and support their differentiation, hold great promises for the development of single step cartilage repair procedures^9^ and have been investigated in preclinical and clinical trials.^10^ However, these *in situ* regeneration techniques most often use a combination of a biomaterial with microfracture. Drilling through the subchondral bone causes bleeding in the defect and thereby allows mesenchymal cell migration and initiation of repair.^11^ Nevertheless, the nature of the cartilage repair tissue is fibrocartilaginous and microfractures have been shown to lead to subchondral cyst formation.^12^ Alterations of the subchondral bone have additionally been shown to impair cartilage repair by several mechanisms, such as upward migration of the subchondral bone into the repair site, formation of intralesional osteophytes and subchondral bone cysts as well as changes of the osseous microarchitecture. Further research is needed to understand the mechanism of action and the efficacy of these cell-free, non-microfracture-associated approaches.

We have previously introduced an adhesive and chondro-inductive hyaluronic acid–derived hydrogel, namely hyaluronic acid–transglutaminase (HA-TG).^13^ HA-TG is able to direct proliferation and chondrogenic differentiation of human cells from several origins: fetal chondroprogenitor cells (hCCs),^14^ infant chondrocytes from polydactyly,^15^ and adult auricular chondrocytes.^16^ Given its fast gelation kinetics and high adhesive properties to cartilage, we proposed HA-TG as a cell carrier for injectable, cartilage engineering applications. Subcutaneous implantation in an ectopic mouse model showed that HA-TG was not permissive to vascularization, promoted chondrogenesis with encapsulated cells and led to the formation of stable cartilage grafts.^15^ It was unknown, though, whether this biomaterial would support cell migration and resist the mechanically challenging joint environment.

Preclinical studies are a key requirement for the clinical translation of new medicinal products.^17,18^ Nevertheless, choosing the most appropriate animal model for the translation of cartilage engineering application remains a challenge.^19,20^ The rabbit model is easy to handle and allows chondral defect creation, but spontaneous regeneration has been reported due to increased chondrocyte metabolic activity and higher cell density in cartilage tissue.^20^ The ovine stifle joint model has been used to investigate a range of orthopedic conditions,^21^ although the experimental setup varies. While spontaneous healing of small chondral defects has been shown to occur in fetal lamb,^22^ critical size chondral defects of 6 mm diameter in skeletally mature sheep do not fully heal and rather lead to the formation of a scar tissue.^17,23^

The aim of this study was to evaluate HA-TG as a biomaterial for cell-free, *in situ* cartilage engineering applications in a clinically relevant, large animal model. For this reason, we chose full thickness, chondral defects in the stifle joint of skeletally mature sheep as a model. The defect model was first refined and the biocompatibility of HA-TG in a collagen scaffold (Optimaix) assessed in a short-term (3 months) study. Then, the potential of this combination of HA-TG gel and collagen scaffold to support cartilage regeneration and prevent adjacent cartilage breakdown was evaluated in comparison with empty defects in a 6-month study.

## Methods

### Hydrogel

*HA-TG*: HA-TG hydrogel precursors, TG/Gln and TG/Lys, were synthetized by substituting carboxylic acid moieties of hyaluronan chains (Lifecore Biomedical, 1.01-1.8 MDa) with reactive glutamine residue (NQEQVSPL-ERCG) and reactive lysine residues (FKGG-ERCG) respectively as previously described.^13,14^ HA-TG precursors were solubilized at 2% (w/v) in sterile filtered Tris buffered glucose solution (glucose 100 mM, CaCl_2_ 50 mM, Tris 50 mM, balanced to pH 7.6). The crosslinking was initiated by adding thrombin (Baxter) and factor XIII (Fibrogammin, CSL Behring) to a final concentration of 12.5 U/mL and 10 U/mL, respectively.

### Collagen Scaffold

Optimaix-3D (1.5 mm in height) is an open porous porcine collagen I/III sponge (containing <30% w/w elastin) produced by a zero-length crosslinking procedure using EDC/NHS chemistry. Optimaix scaffolds were punched into 6mm-diameter cylinders and placed in the defect prior to addition of HA-TG hydrogels in order to improve the stability of the HA-TG gel.

### Animal Medication and Surgery

Female, skeletally mature (2-3 years old) healthy Maedi-Visna negative Swiss Alpine sheep were used for the experiments from the Musculoskeletal Research Unit’s own herd, after being acclimatized for 7 days. Three sheep were randomly chosen by hand from the herd for the 3-months survival group and 8 sheep for the 6-months survival group. The *in vivo* experiments were conducted at the Musculoskeletal Research Unit according to Swiss laws for animal welfare and approved by the local governmental authorities (Kantonales Veterinäramt Zürich, Switzerland, No. ZH193/15).

The sheep were sedated by intramuscular injection of 0.1 mg/kg xylazine and 0.01 mg/kg buprenorphine. Anesthesia was then induced intravenously via a jugular catheter with 0.1 mg/kg midazolam, 3 mg/kg ketamine, and 0.4 to 0.9 mg/kg propofol. Following laryngeal desensitization with lidocaine spray, the trachea was intubated. Anesthesia was maintained with an intravenous constant-rate infusion of 1 mg/kg/h propofol in combination with 1% to 3% isoflurane in oxygen. Epidural anesthesia was applied with 0.1 mg/kg morphine diluted in sterile 0.9% NaCl to a total volume of 2 mL. Intravenous penicillin (30,000 IU/kg, Streuli Pharma) and gentamycin (4 mg/kg, Vetagent, MSD Animal Health Care) were administered on the day of surgery as preoperative antibiotic prophylaxis and for 4 days thereafter. A booster for tetanus (3,000 IU/sheep, MSD Animal Health Care) was administered subcutaneous on the day of surgery. Regarding analgesia, carprofen (4 mg/kg) was administered intravenously on the day of surgery and for 4 days thereafter. Buprenorphine (0.01 mg/kg) was applied 3 times after surgery, every 4 to 6 hours.

**Figure 1.**
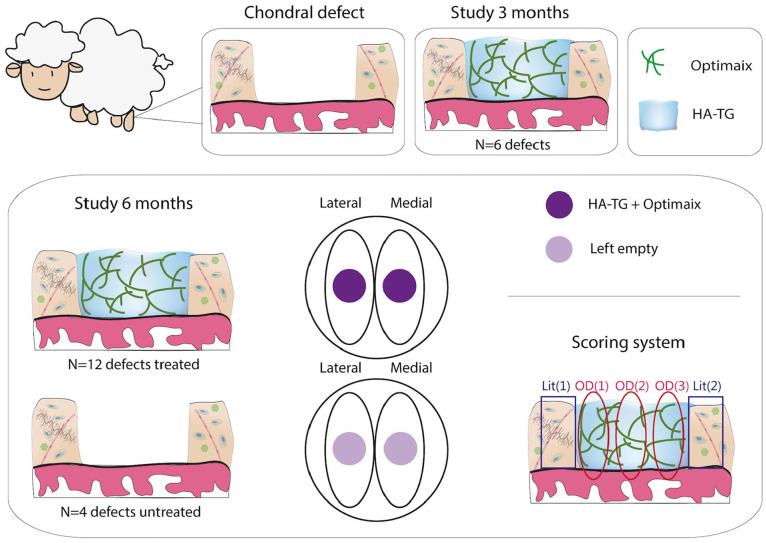
Schematic of the project. Chondral defects were surgically prepared in ovine stifle joints. In the 3-month study, 6 defects (2 defects per condyle, 1 condyle per animal) were filled with hyaluronic acid–transglutaminase (HA-TG) and Optimaix. In the 6-month study (bottom illustrations), 12 defects (1 defect per condyle, 2 condyles per animal, as depicted in the central illustration, where defects and treatment distributions are shown) were either treated with HA-TG and Optimaix (*n* = 12 defects) or left untreated (*n* = 4). Histological sections were scored with a modified O’Driscoll (OD) and a modified Little (Lit) score, and the final score consists of the average of the 3 (OD) or 2 (Lit) area scored (bottom right illustration).

A para-patellar approach was chosen with the stifle joint in maximal flexion to access the weightbearing area of the femoral condyles (Supplemental Figure 1). Full thickness cartilage defects (6 mm in diameter) were created in the weightbearing area in the medial and lateral condyles of the distal femur of one hind limb, with the other hindlimb left intact. Operated limbs (left or right) were alternated from one animal to the next. For the 3-month study, 2 defects per condyle were created. The defect site was marked using a biopsy punch. Cartilage was then removed from the defect site using a scalpel down to the calcified cartilage layer, and the last step of the defect debridement was done using a drill burr. For the 6-month study, 1 defect per condyle was created. Defects were created with a device provided by Xiros Ltd (Supplemental Figure 2). In all treated defects (6 defects in 3 sheep in the 3-month study and 12 defects in 6 sheep in the 6-month study), Optimaix scaffolds were placed in the defect and HA-TG was injected on top. HA-TG was allowed to crosslink for 10 minutes before closing the joint. The 4 defects in 2 additional sheep in the 6-month study were left empty as controls (**Fig. 1**). A cast was placed on the operated limb for the 5 weeks postoperatively to minimize stifle joint movement and animals were placed in a suspension net for 3 weeks after surgery to reduce, but not eliminate, loading on the stifle joint. Following cast removal, a soft bandage was placed over the wound for 4 days and then removed.

Sheep were checked twice daily and their appetite, posture, alertness, pain, respiration, and weightbearing were evaluated and scored. 3 or 6 months following surgery, sheep were taken to slaughter. A captive bolt was used to render them instantly unconscious. Death was confirmed by the absence of corneal reflex and the knees dissected thereafter.

### Macroscopic Evaluation, Histology, and Histological Scoring

After sacrifice, defect sites were macroscopically qualitatively assessed for the extent and quality of the defect filling as well as the health of the surrounding cartilage. Photos were taken in order to measure the surface covered by repair tissue, as a percentage of the total surface of the defect. The quantification was done using Image J. Then, osteochondral blocks for each condyle containing both the defect areas, surrounding cartilage and subchondral bone, were prepared and fixed in 4% formalin. Following MMA (methyl methacrylate) embedding, each condyle block was cut in half, following a proximal-distal plane transecting the middle of the defects. One half of each block was then used to create one ground section (400-600 µm), starting from the middle of the defect, and was surface stained with toluidine blue. The second half of the block was trimmed then used to cut 3 thin sections (5 µm), also starting from the middle of the defect. The thin sections produced were stained with toluidine blue, safranin-O/fast green/hematoxylin and von Kossa/McNeal.

Toluidine blue– and safranin-O–stained sections were blind-scored independently by 2 individuals using 3 scores to semiquantitatively evaluate repair tissue quality (modified O’Driscoll score), adjacent cartilage state (adapted Little score), and subchondral cysts (custom-made score). Modified O’Driscoll score was used to assess the quality of the repair tissue in the defects (Supplemental Table 1),^24^ since this score was shown to have a low interobserver variability.^25^ Analyzed criteria included bonding of new tissue to adjacent cartilage, interterritorial and pericellular glycosaminoglycan (GAG) staining, cellularity in the defects and cell morphology. For criteria evaluated at both the distal and proximal edges of the defect, an average was calculated in order to compute the total summed score, which was used for statistical analyses (**Fig. 1**). The total modified O’Driscoll score ranged from 0 (hyaline tissue) to 20 (fibrous tissue or no repair tissue). The quality of the cartilage adjacent to the defect on both sides was scored using an adapted score first described by Little *et al*.^26^ (Supplemental Table 2). Criteria included the structure of the tissue, its cellularity, the amount of the GAGs present and the integrity of the subchondral bone. Scoring was done for both the distal and proximal edges of the defect and the final score was calculated as the sum of the averages of the individual criteria. The total Little score ranged from 0 (normal cartilage) to 29 (severely abnormal or absent cartilage). A cyst score was introduced, including criteria of size, isolation from the synovial fluid, the nature of the filling tissue, the activity of the bone and nonfibrotic tissues (Supplemental Table 3). It ranged from 0 (no cyst) to 19 (severe cyst with low probability of resorption). The total score, calculated as the sum of the O’Driscoll, Little, and cyst scores, ranged from 0 (best) to 68 (worst).

### Statistical Analysis and Raw Data

The 3-month study included *n* = 6 defects in 3 sheep in total for analysis as an exploratory proof of concept to generate sufficient data for refinement. The 6-month study included *n* = 12 treated defects in 6 sheep and *n* = 4 untreated control defects in 2 sheep. The reduced sample size for untreated defects was chosen in accordance with 3R (replacement, reduction, and refinement) guidelines, as 6 mm full thickness cartilage defects were expected to be critical size defects and for which, in our previous experience, spontaneous repair does not occurs, thus providing a “no intervention” control allowing to assess the potential effect of a gel and collagen scaffold treatment. All data from the aforementioned defects/animals was included in the study analyses. All raw data are kept in the MSRU and ETHZ digital and paper data archives. The GraphPad Prism was used for all statistical operations. Comparison of results was carried out by *t* test (or Kruskall-Wallis if normality of distribution was not found by the Shapiro-Wilk test) and analysis of variance (ANOVA) using Tukey’s multiple comparison *post hoc* test for significance. The threshold for statistically significant difference was set at *P* = 0.05.

## Results

### Results After 3 Months Highlight the Biocompatibility and the Capacity of Acellular Gels to Be Colonized by the Host Cells

The surgical procedure presented no complications. All animals tolerated the surgery well and showed no persistent lameness or abnormal behavior. There were no clinical abnormalities noted, beyond those expected after surgery.

**Figure 2.**
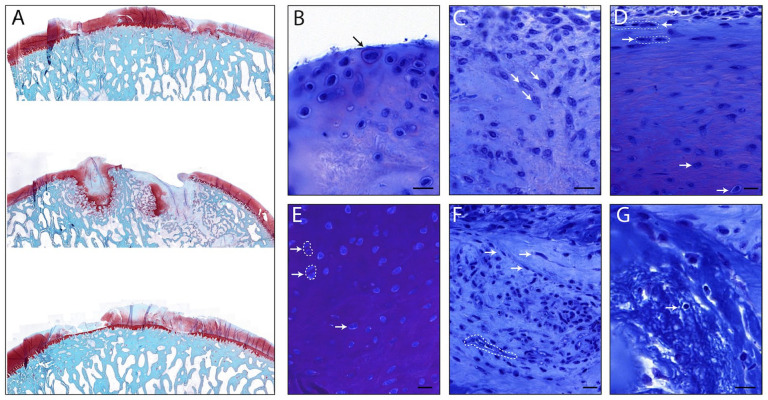
Defects filling, 3 months after implantation. (**A**) Safranin-O staining of the defects, coronal cut of the whole condyles. Scale bar: 1 mm. (**B-G**) Toluidine blue staining for the study of cells phenotype in defects after implantation of acellular hyaluronic acid–transglutaminase (HA-TG) gels in chondral defects. Scale bars: 20 µm. (**B**) The black arrow points at a large mesenchymal cell surrounded by a glycosaminoglycan (GAG)-rich pericellular region. (**C**) White arrows point at cells whose nucleus displays area of dense chromatin, visualized by dots of darker toluidine blue staining. (**D**) Flattened elongated cells aligned in rows at the surface of the defect filling are circled in white dotted lines, and rounder cells toward the deeper zone of the defect filling are pointed to with white arrows. (**E**) Cell doublets are circled in white dotted lines and highlighted with white arrows. The white arrow alone points at a cell undergoing mitosis. (**F**) A neovessel is circled with a white dotted line and fibroblastic, elongated cells are pointed at with white arrows. (**G**) The white arrow points at a lymphocyte.

After 3 months, high variability was observed in the quality of the repair tissue. As evidenced by histological staining, GAG deposition in the defects was abundant throughout the defect area in the best cases, but only at the border regions to native cartilage in the worst cases (**Fig. 2A**). The gels were colonized by cells from the host. Remarkably, cell morphology was chondrocyte-like, with a large size (~20 µm) and GAG deposition in territorial region (**Fig. 2B**, black arrow). These cells appear to be active, considering the presence of regions of highly condensed chromatin in the nuclei (**Fig. 2C**, white arrows). In addition, when repair tissue was present in the cartilage defect, the structural cell organization resembled the one in native articular cartilage, with elongated cells in the superficial layer and more round cells in the deeper zones (**Fig. 2D**). There was evidence of cell proliferation, as seen by the frequent presence of doublets within the repair tissue (**Fig. 2E**, doublets in dotted lines, mitotic cell pointed with a white arrow). In some cases, there were fibroblastic, elongated cells (**Fig. 2F**, white arrows), and very rarely, some neovascularization (**Fig. 2F**, in dotted line). Importantly, no acute inflammatory response to the biomaterial implantation was observed. There were no major invasion of lymphocytes or other inflammatory cells (such as polymorphonuclear cells) in the three quantifiable samples (the samples where a cyst formed and where no repair tissue was visible were excluded). Indeed, only 2 lymphocytes could be spotted in the sections analyzed (smaller cell with dense nucleus, **Fig. 2G**, white arrow).

It appeared that the creation of the defect was a cause of variability in the quality of the repair, since the tidemark was violated for some defects (**Fig. 2A**, middle) and this resulted in cyst formation. In order to better control the depth of the defect and avoid production of heat in the following 6-month study, surgical creation of the defect was made by replacing the drill by a custom-made, hand-operated tool. The tool consisted of a circular hollow blade holder held in place on the condyle and within which a concave blade was turned (Supplemental Figure 2) until the calcified cartilage was reached.

Additionally, we had observed the collapse of the cartilage tissue in between 2 defects in preparatory studies and in 1 out of 3 condyles presented in **Fig. 2A** (middle), possibly due to defect proximity. Consequently, for the 6-month study, only 1 defect per condyle was made.

### Macroscopic Evaluation at 6 Months Shows Variable Results

In the 6-month study, treatment with HA-TG + Optimaix was compared with empty defects, as recommended by the International Cartilage Repair Society,^27^ to control for spontaneous regeneration.

**Figure 3.**
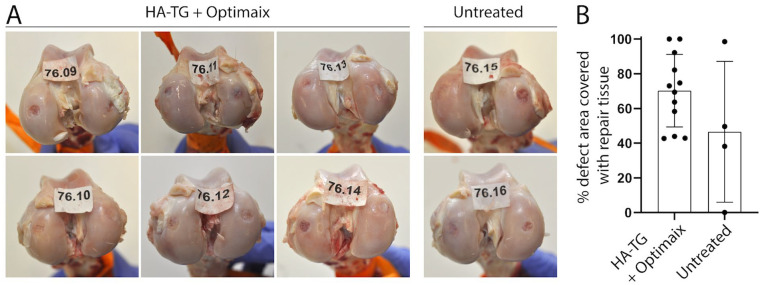
Macroscopic assessment of cartilage repair 6 months after implantation. (**A**) Pictures of condyles after explantation (labels indicate the sheep number given to perform a blind scoring). (**B**) Quantification of area covered with white repair tissue.

The macroscopic evaluation showed that the filling was mostly partial, both in terms of height and surface coverage (**Fig. 3A**). Quantification of the defect area covered with repair tissue showed a surface coverage ranging from 40% to 100% in treated defects (mean 70.2% ± 21%). Empty defects showed highly variable coverage with a mean defect area covered of 46.6% ± 40.6%, and values ranging from 0% to 98.5% (**Fig. 3B**).

### Histological Scorings of Adjacent Tissue Preservation and Defect Filling Quality Suggest a Protective Effect of HA-TG on Adjacent Cartilage

**Figure 4.**
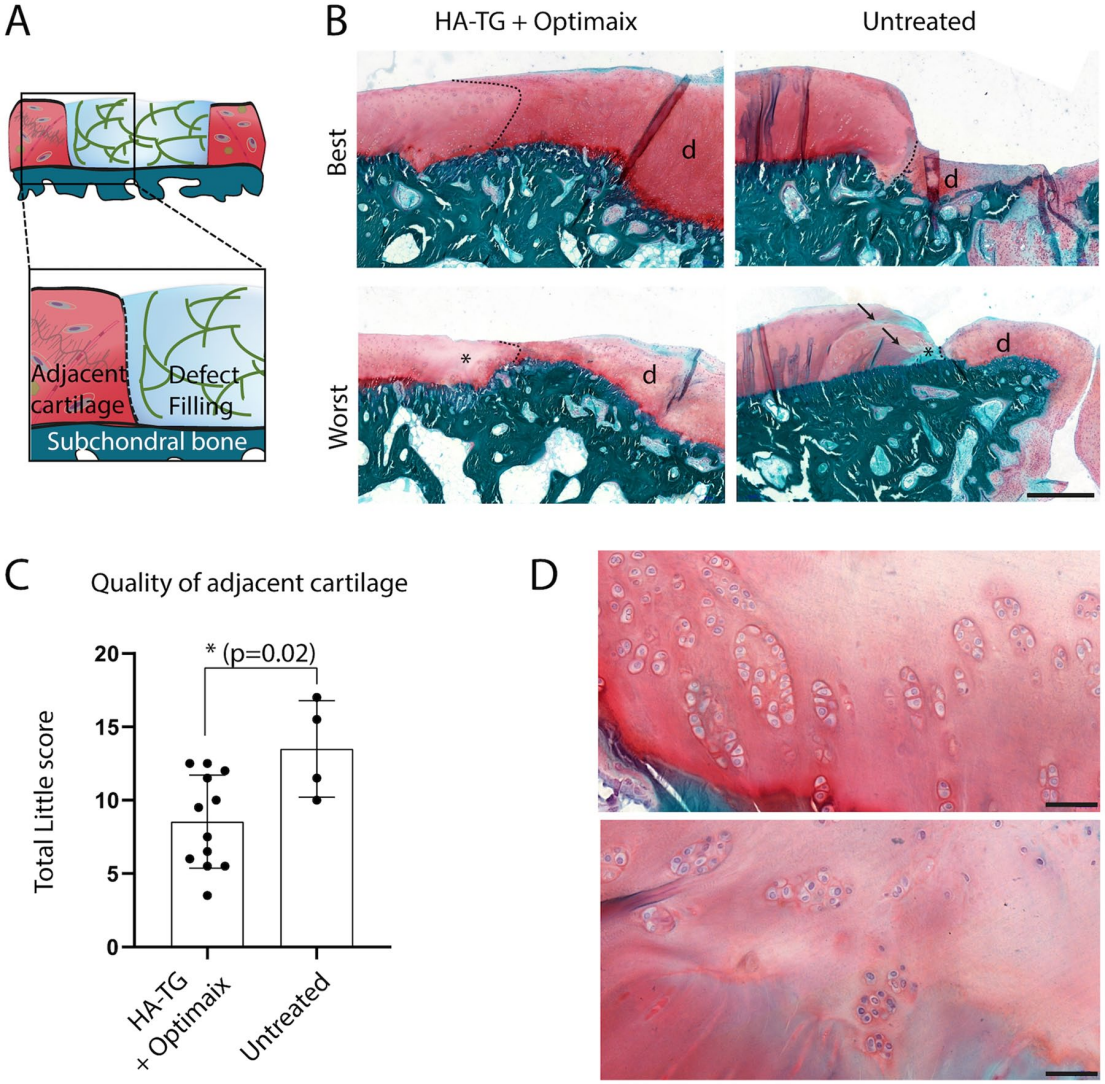
Effect of defect treatment on maintenance of surrounding cartilage, 6 months after surgery (**A**) Schematic of the structures shown in the close-up images in (**B**). Representative images of best- and worst-scored adjacent cartilage from safranin-O–stained sections. All images were taken at the same magnification. The separation between the native cartilage tissue (left side) and the defect (indicated with the letter “d”) is drawn with dotted black lines. Asterisks indicate areas with fainter safranin-O staining. Arrows point at clefts. Scale bar: 500 µm. (**C**) Plotted Little scores (*n* = 12 for defects treated with hyaluronic acid–transglutaminase (HA-TG) + Optimaix, *n* = 4 for untreated defects). (**D**) Representative images of cell clusters in the surrounding cartilage. Scale bar: 50 µm.

A significant improvement in treated defects was observed in terms of preservation of the adjacent cartilage quality when compared to empty controls, as illustrated in **Fig. 4A** and **B** and semiquantified with the Little score (**Fig. 4C**, mean Little score was 8.5 ± 3.2 in treated defects vs. 13.5 ± 3.3 in untreated defects, *P* = 0.02). Of note, we observed some cell clusters in GAG-rich regions of the adjacent cartilage, both in treated and empty defects. This corresponded to a score of 4 in the “Cell cloning” section of the Little score (Supplemental Table 1), which was found in 12 sites (left and/or right side of defects) in treated defects (50%) versus 4 sites in empty defects (50%) (**Table 1**).

**Table 1.** Overview of Individual Scores in Adjacent Cartilage Quality (Adapted Little Score).

Treatment	Animal	Condyle	Structure		Tidemark		ECM GAG		Pericellular GAG		Cellularity		Cloning		Little Score Total^a^
			Left	Right	Left	Right	Left	Right	Left	Right	Left	Right	Left	Right	
HA-TG + Optimaix	76.09	Med	4	7	0	0	1	1	1	1	1	1	4	4	12.5
	76.09	Lat	1	0	0	1	1	1	0	1	1	1	2	2	5.5
	76.10	Med	0	0	0	0	0	1	1	1	0	0	2	2	3.5
	76.10	Lat	4	0	1	1	0	1	1	1	1	1	3	1	7.5
	76.11	Med	0	1	1	1	2	1	1	0	2	0	1	1	5.5
	76.11	Lat	2	5	0	3	0	1	1	1	2	1	4	4	12
	76.12	Med	0	5	0	0	2	1	1	1	2	3	4	0	9.5
	76.12	Lat	0	1	0	0	0	3	1	1	1	2	2	2	6.5
	76.13	Med	1	6	0	1	0	2	2	1	1	1	4	1	10
	76.13	Lat	1	7	1	1	2	1	0	1	2	1	4	4	12.5
	76.14	Med	5	1	1	1	1	1	1	1	1	2	4	4	11.5
	76.14	Lat	0	0	1	0	0	0	1	1	0	1	4	4	6
Empty	76.15	Med	5	0	0	3	0	2	1	2	1	2	4	3	11.5
	76.15	Lat	8	7	0	0	4	1	2	1	3	1	1	3	15.5
	76.16	Med	0	7	0	1	1	1	1	1	1	1	2	4	10
	76.16	Lat	1	8	3	1	4	2	3	1	2	1	4	4	17
Maximum score			10	10	3	3	4	4	4	4	4	4	4	4	29
Minimum score			0	0	0	0	0	0	0	0	0	0	0	0	0

ECM = extracellular matrix; GAG = glycosaminoglycan; HA-TG = hyaluronic acid–transglutaminase; Lat = lateral; Med = medial.

a The total score indicates an average of right and left regions of interest in each sample. A schematic representation of histological evaluation sites within each defect filling is shown in **Figure 1**.

**Figure 5.**
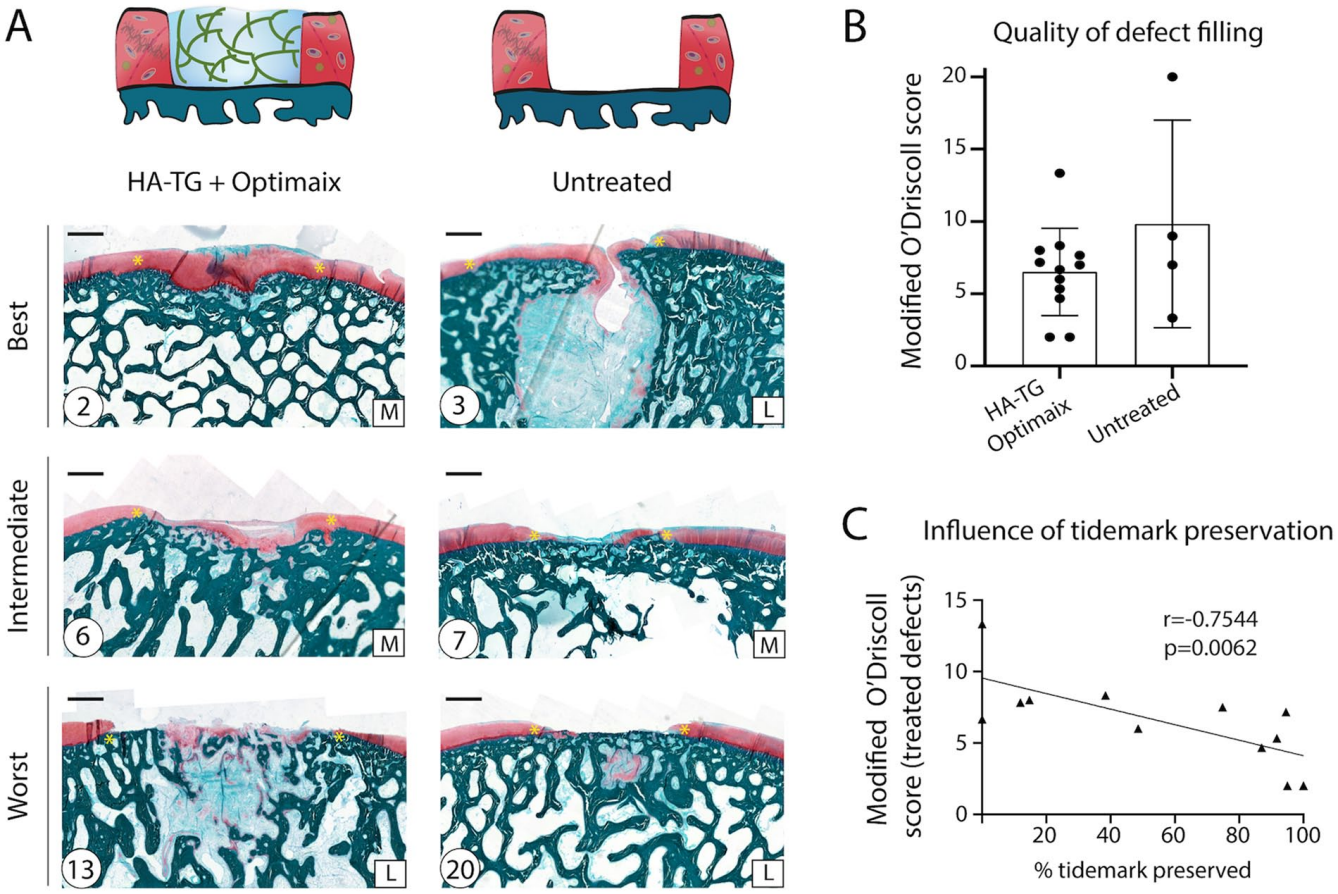
Defects filling, 6 months after implantation. (**A**) Safranin-O staining of the defects (*n* = 12 for defects treated with hyaluronic acid–transglutaminase (HA-TG) + Optimaix, *n* = 4, for untreated defects, schematics of the condition on top of each column). “L” and “M” on the bottom right stand for “Lateral” and “Medial” condyle, respectively. “Best” and “Worst” refer to the O’Driscoll scores, which are provided on the bottom left corner, circled. Yellow stars indicate the defect edges. Scale bars: 1 mm. (**B**) Total modified O’Driscoll score for all conditions. (**C**) Plotted O’Driscoll scores from treated defects, as a function of the tidemark preservation (measured proportion of the defect length where calcified cartilage is visible and clearly separated from noncalcified cartilage on the histological sections).

As observed after 3 months, the repair tissue displayed high cellularity. The quality of the repair tissue at 6 months in defects treated with HA-TG with Optimaix was not significantly different than in untreated defects, partly due to the highly variable scores of empty defects (mean O’Driscoll scores: 6.6 ± 3.0 versus 9.8 ± 7.2, respectively, *P* = 0.2, **Fig. 5A** and **B**). However, the histological GAG staining suggested a poorer tissue repair quality, since the best sample from the empty defect group actually presented a cyst filled with fibrous tissue opened to the synovial cavity. Importantly, the quality of the filling positively correlated with the percentage of the tidemark preserved (**Fig. 5C**).

### Evaluation of the Subchondral Bone Emphasizes Its Crucial Role in Cartilage Repair

At 6 months, the occurrence of cysts in the subchondral bone was observed on Von Kossa–stained sections (4/12 treated defects, 2/4 in untreated defects, Supplemental Figure 3).

**Figure 6.**
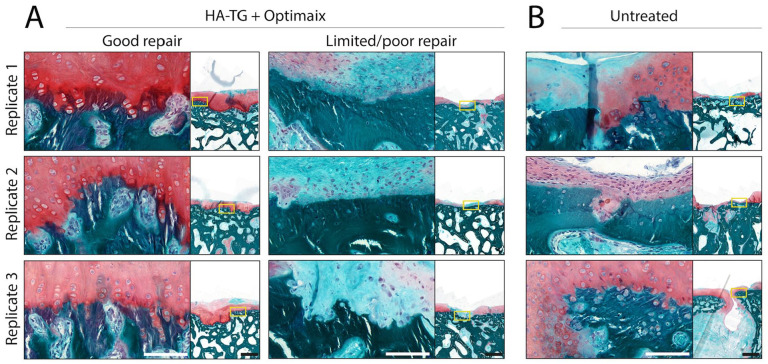
Cell migration from the subchondral bone into cartilage defects, 6 months after surgery. (**A**) Close up on safranin-O–stained sections of defects treated with hyaluronic acid–transglutaminase (HA-TG) + Optimaix, where no cyst was observed. Close-up images are shown next to an image of whole view on the defect (yellow rectangles indicate where the close-up was taken from). Three representative images of good (left) and limited/poor (right) repair tissue are shown. The 3 representative “replicates” are sections from 3 different defects, taken from 3 different sheep. (**B**) Close-up on safranin-O–stained sections of empty defects, where some repair tissue was present (Note: No repair tissue was observed in the fourth defect). Scale bars: 100 µm (close-ups) and 1 mm (whole defect).

The high cellularity in defect filling (score 0 in the “cellularity” section of the O’Driscoll score, found, for 8 treated defects, in all—left, right, and center—scored area, and in at least 1 scored area in the 4 remaining defects; see **Table 2**), together with the correlation between defect filling quality and preservation of the tidemark, suggest the migration of cells from the subchondral bone into the defect through the calcified cartilage layer. This was indeed visible in HA-TG + Optimaix–treated defects displaying excellent repair (**Fig. 6**, left column), where columns of chondrogenic cells surrounded by a positive GAG staining can be observed throughout the calcified cartilage layer. In case of poor repair, this feature was not present (**Fig. 6**, middle column). In untreated samples, chondrogenic cells migration could be seen in one defect, although the whole repair was not promising due to the presence of an open cyst (**Fig. 6**, right column). These observations further strengthen the crucial role of the subchondral bone in promoting hyaline-cartilage regeneration.

**Table 2.** Overview of Individual Scores for Repair Quality (Adapted O’Driscoll Score).

Treatment	Animal	Condyle	Tidemark	Coverage	Bonding		Thickness			GAG			Cellularity			Cell Type			O’Driscoll Total^a^
					Left	Right	Left	Right	Center	Left	Right	Center	Left	Right	Center	Left	Right	Center	
HA-TG + Optimaix	76.09	Med	2	0	0	0	2	3	3	0	2	2	0	1	0	1	2	2	8
	76.09	Lat	2	0	1	1	1	3	3	0	0	3	0	0	0	1	1	2.5	7.8
	76.10	Med	1	0	0	0	0	0	0	0	0	0	0	0	0	1	1	1	2
	76.10	Lat	2	0	0	0	3	0	3	0	1	3	0	0	0	1	1	2	6.7
	76.11	Med	2	0	0	0	0	0	2	0	2	3	0	0	0	1	2	2	6
	76.11	Lat	2	0	0	0	0	1	2	1	0	0	1	0	0	1	1	1	4.7
	76.12	Med	2	0	0	0	3	0	3	3	1	3	0	0	0	3	1	2	8.3
	76.12	Lat	1.5	0	0	0	3	0	0	3	0	3	1	0	3	3	0	2	7.5
	76.13	Med	2	0	0	1	2	1	3	1	0	3	0	0	0	1	1	2	7.2
	76.13	Lat	2	0	0	0	2	2	0	0	0	2	0	0	0	1	1	2	5.3
	76.14	Med	1	0	0	0	0	0	0	0	0	0	0	0	0	1	1	1	2
	76.14	Lat	2	2	1	1	3	2	3	3	3	3	2	0	0	2	2	2	13.3
Empty	76.15	Med	2	0	0	0	2	2	2	1	1	3	0	0	0	1	1	2	7
	76.15	Lat	2	3	2	2	3	3	3	3	3	3	3	3	3	4	4	4	20
	76.16	Med	2	0	0	0	3	3	3	1	3	3	0	0	0	1	2	2	9
	76.16	Lat	1	1	0	0	0	2	0	0	1	0	0	0	0	1	0	0	3.3
Maximum score			2	3	2	2	3	3	3	3	3	3	3	3	3	4	4	4	20
Minimum score			0	0	0	0	0	0	0	0	0	0	0	0	0	0	0	0	0

GAG = glycosaminoglycans; HA-TG = hyaluronic acid–transglutaminase; Lat = lateral; Med = medial.

a The total score indicates an average of right, left, and center regions of interest in each sample. A schematic representation of histological evaluation sites within each defect filling is shown in **Figure 1**.

## Discussion

Considering the drawbacks of autologous chondrocyte implantation and microfracture, *in situ* cartilage regeneration holds great promise for a single-step, less invasive procedure. In this study, we showed the potential of the combination of a collagen scaffold (Optimaix) with HA-TG, an enzymatically crosslinked hyaluronan-based hydrogel, as an acellular tissue engineering approach for *in situ* regeneration. Indeed, HA-TG in combination with a collagen I/III sponge (Optimaix) can adhere to the surrounding tissues which is paramount to long-term success of cartilage repair strategies.^28,29^ The material could also help recruit and retain mesenchymal cells, based on the observation that defect fillings were hypercellular (8 defects scored 0 for the “cellularity” section of the modified O’Driscoll score, **Table 2**) and that many scored areas highlighted the presence of incompletely differentiated mesenchyme cells (grade 2 in the “cellular morphology” section of the modified O’Driscoll score, found in 11 of the 36 scored area, **Table 2**). This mesenchymal cell recruitment and subsequent stimulation of their chondrogenesis^14^ are requirements inherent to *in situ* regeneration.^30^ At 6 months, migration of chondrogenic cells from the subchondral bone was clearly visible in successfully regenerated cartilage defects and treatment with HA-TG + Optimaix led to better preservation of adjacent cartilage compared to empty defects.

Three months postsurgery, a high variability could be observed at both macroscopic and microscopic levels. A previous study on chondral defect repair in sheep already reported that the highest individual differences between animals were observed between weeks 8 and 12 postsurgery.^11^ Other studies reported reduced differences at the 6- and 12-month time points.^31,32^ It has been argued that the wide range of cartilage thicknesses within an ovine stifle joint, ranging from 0.7 to 1.2 mm depending on the region in the weight bearing area of the condyle, can cause some variability in repair outcomes.^20^ In addition, some variability is linked to the mechanical environment,^33^ which notably differs between lateral and medial condyles. The small number of samples and the influence of defect location limit generalization; however, HA-TG in collagen displayed good histological outcome, with the presence of cells within the defect filling and GAG deposition.

We found histological evidence of mesenchymal-like cells found in the defect filling, which may have possibly migrated from the subchondral bone through the tidemark, in treated defects displaying an intact tidemark. In other words, we could show in defects treated with HA-TG + Optimaix that the presence of an intact subchondral bone provided an environment favorable to mesenchymal cells invasion, which theoretically might occur via the CD44 receptor of these cells,^34^ and cartilaginous matrix deposition. More work will be needed to understand the physicochemical cues that promoted cell migration, and how HA-TG and the collagen scaffold is remodeled by these cells over time. Of note, it cannot be excluded that the invading cells come from several tissues, since mesenchymal cells from the synovium have also been shown to migrate into chondral defects in rabbits and minipigs.^35^ The ability of cells from the subchondral bone to migrate through the calcified cartilage layer has been described *in vivo* in small animal models^34^ and *in vivo* in a sheep model using photo-oxidized cartilage plugs for mosaicplasty via the formation of cones in the tidemark on surgical mechanical disruption.^36^ In this proof-of-concept study, we could not determine whether the treatment of defects with HA-TG+Optimaix, which adheres to the surrounding tissues and supports cell migration, led to better-quality repair tissue, compared with nontreated defects. This is partly due to the variability of results among nontreated control samples and the small sample size in this group which, although in line with 3R principles, exhibited a surprisingly higher percentage of coverage than expected for untreated critical size defects. Indeed, one of the empty defects showed good defect filling despite the presence of an open cyst. Complete coverage of untreated defects with GAG-rich tissue was already reported but not discussed in previous studies.^37,38^ These observations require rethinking of “critical size defects” definition and standardizing defects creation in sheep studies. Furthermore, designing a study with a reference treatment group (e.g., microfracture) instead of a nontreated control may provide valuable insights for clinical translation, whilst staying in line with 3R principles of animal experimentation. Further work will be needed to determine the mode of action of HA-TG + Optimaix.

Our study showed that chondrocyte proliferation was taking place in the adjacent cartilage at the edge of the defects, with the presence of many cell clusters especially in GAG-rich regions. This suggests that the host’s chondrocytes at the edges of the defect also play a vital role in driving the regeneration of the tissue. It was hypothesized that healing of osteochondral defects also happens by neocartilage formation in the cartilage adjacent to the defect.^11^ Neocartilage is then deposited from the sides into the cartilage defect and provides an appropriate conduit for further cartilage production and contributes to the secretion of local trophic factors to induce differentiation of undifferentiated cells into chondrocytes.^39^ Our study implies that cell clustering and proliferation at the edges of the defects could be important for further matrix deposition and repair within the defect, which is in contradiction to the accepted view that clusters (as is seen in many scores) are tied to degeneration and “failed repair” in the context of osteoarthritis.^40^ There is likely a spectrum of processes these clusters may be linked to, depending on their surroundings and stage of repair. A mild degradation of the cartilage interface was observed in treated defects at 6 months, which is expected due to the mechanical stress resulting from the height variation in the defect area, as well as the lowered chondrocyte viability associated to the surgical preparation of the defects.^28^ Yet, HA-TG + Optimaix semiquantitatively better preserved the adjacent cartilage as compared with empty defects, suggesting that the implanted biomaterials, via the prevention of the nearby cartilage degradation, support tissue regeneration. Indeed, studies have shown that high molecular weight hyaluronan is associated with anti-inflammatory and immunosuppressive properties that can protect from ECM degradation and in turn from chondrocytes apoptosis, which can explain the beneficial effect of hyaluronan intra-articular injections. It can also be hypothesized that HA-TG + Optimaix prevented the mechanical disruption of the surrounding cartilage deriving from the height gap. Of note, the study design does not allow to determine the contribution of the commercial collagen scaffold alone; this group could not be added due to the fact that the collagen scaffold Optimaix could not stay in the defect without being fixated, as observed with preliminary cadaver tests (data not shown). Additionally, in clinical practice, other collagen scaffolds are usually used in the context of MACI, but not alone.

The 6-month study provided a scientific evidence to the commonly acknowledged necessity to preserve the tidemark while debriding the defect. With higher number of animals in the 6-month study, we could highlight a correlation between tidemark preservation and quality of cartilage tissue repair. In case of tidemark disruption during defect creation, cysts can form as described in the microfracture procedure^12^ and the tissue regeneration can thus be impaired because of the experimental setting rather than the inefficiency of the tested strategy. These facts call for a standardization of defect making, as suggested by Schwarz *et al*.^41^ This standardization is not only important when testing regeneration solutions in preclinical studies but also when a surgeon is preparing a site clinically, for the quality of the repair. The status of the subchondral bone below a cartilage defect correlates with the success rate of clinical regenerative techniques^42,43^ and it is becoming apparent that an intact subchondral bone is necessary for the success of articular cartilage treatments.^44^ Particularly, advancement of the subchondral plate toward the bone has raised concern. In our study, we observed such subchondral plate advancement in 3 of the 12 treated defects (25%) and in 2 of the 4 defects left empty (50%). The seemingly beneficial effect of HA-TG + Optimaix with regard to subchondral bone advancement should be confirmed with a larger study. Of note, the proportion of defects displaying subchondral plate advancement is close to what was reported 12 months after autologous chondrocyte implantation (18%). Although the clinical relevance of this advancement is not clear, particular attention should be brought on the progression of the proportion of defects presenting this feature in longer-term studies. Given the type of defect filling observed in this study, both a biological role and a biomechanical role (more closely linked to cyst formation) can be highlighted for an intact tidemark.

Von Kossa stainings showed the active remodeling of the subchondral bone, and the presence of cysts in a third of the treated defects. The uneven heights between the defect and the surrounding cartilage can lead to cyst formation, as reported in a mosaicplasty study,^45^ and to adjacent cartilage degeneration. Optimaix was selected since it improved HA-TG compressive modulus *in vitro* without altering HA-TG adhesion to cartilage, which relies on the crosslinking of the HA backbone to the proteins present in the surrounding cartilage, *in vitro* and *in vivo* (Supplemental Figure 4). Despite mechanical reinforcement, the height of defects filled with HA-TG after 6 months was generally lower than the height of the adjacent cartilage. Further work is ongoing to develop a stronger scaffold, whose thickness is higher than that of articular cartilage, in order to promote mechanical stimulation on compression.

Finally, in this study, medial and lateral stifle compartments were assumed to act independently, based on the authors’ historical observations in experimental stifle cartilage defects in sheep, particularly with regard to defect coverage, defect filling quality and surrounding cartilage quality. Statistical analyses were therefore conducted by pooling medial and lateral defect data, generating 2 data points per animal. It is possible that stifle compartments within the same animal may not behave independently and therefore would require an alternate statistical analysis. An alternate analysis was therefore attempted with the same data sets reported above, but separating datapoints from medial and lateral compartments, thereby using only one datapoint per animal in a comparative cohort (Supplemental Figure 5). The same outcomes described for defect coverage, defect filling quality, and surrounding cartilage quality were also observed using the alternate statistical analysis, with differences in surrounding cartilage quality detectable in the lateral compartments, but only showing a trend in the medial compartment. Overall, such an alternate analysis would be more robust with additional datapoints, especially in the untreated group.

In conclusion, we provide a proof-of-concept of HA-TG biocompatibility and capacity to preserve adjacent cartilage, thus providing a favorable environment for the generation of a neocartilage tissue, in a clinically relevant, large animal model. Our study paves the way to a larger preclinical safety study with more sheep and longer duration before considering entering the phase I clinical trial in humans.

## Supplemental Material

sj-docx-1-car-10.1177_1947603521989417 – Supplemental material for Combination of a Collagen Scaffold and an Adhesive Hyaluronan-Based Hydrogel for Cartilage Regeneration: A Proof of Concept in an Ovine Model Click here for additional data file. Supplemental material, sj-docx-1-car-10.1177_1947603521989417 for Combination of a Collagen Scaffold and an Adhesive Hyaluronan-Based Hydrogel for Cartilage Regeneration: A Proof of Concept in an Ovine Model by Clara Levinson, Emma Cavalli, Brigitte von Rechenberg, Marcy Zenobi-Wong and Salim E. Darwiche in CARTILAGE

sj-tif-2-car-10.1177_1947603521989417 – Supplemental material for Combination of a Collagen Scaffold and an Adhesive Hyaluronan-Based Hydrogel for Cartilage Regeneration: A Proof of Concept in an Ovine Model Click here for additional data file. Supplemental material, sj-tif-2-car-10.1177_1947603521989417 for Combination of a Collagen Scaffold and an Adhesive Hyaluronan-Based Hydrogel for Cartilage Regeneration: A Proof of Concept in an Ovine Model by Clara Levinson, Emma Cavalli, Brigitte von Rechenberg, Marcy Zenobi-Wong and Salim E. Darwiche in CARTILAGE

sj-tif-3-car-10.1177_1947603521989417 – Supplemental material for Combination of a Collagen Scaffold and an Adhesive Hyaluronan-Based Hydrogel for Cartilage Regeneration: A Proof of Concept in an Ovine Model Click here for additional data file. Supplemental material, sj-tif-3-car-10.1177_1947603521989417 for Combination of a Collagen Scaffold and an Adhesive Hyaluronan-Based Hydrogel for Cartilage Regeneration: A Proof of Concept in an Ovine Model by Clara Levinson, Emma Cavalli, Brigitte von Rechenberg, Marcy Zenobi-Wong and Salim E. Darwiche in CARTILAGE

sj-tif-4-car-10.1177_1947603521989417 – Supplemental material for Combination of a Collagen Scaffold and an Adhesive Hyaluronan-Based Hydrogel for Cartilage Regeneration: A Proof of Concept in an Ovine Model Click here for additional data file. Supplemental material, sj-tif-4-car-10.1177_1947603521989417 for Combination of a Collagen Scaffold and an Adhesive Hyaluronan-Based Hydrogel for Cartilage Regeneration: A Proof of Concept in an Ovine Model by Clara Levinson, Emma Cavalli, Brigitte von Rechenberg, Marcy Zenobi-Wong and Salim E. Darwiche in CARTILAGE

sj-tif-5-car-10.1177_1947603521989417 – Supplemental material for Combination of a Collagen Scaffold and an Adhesive Hyaluronan-Based Hydrogel for Cartilage Regeneration: A Proof of Concept in an Ovine Model Click here for additional data file. Supplemental material, sj-tif-5-car-10.1177_1947603521989417 for Combination of a Collagen Scaffold and an Adhesive Hyaluronan-Based Hydrogel for Cartilage Regeneration: A Proof of Concept in an Ovine Model by Clara Levinson, Emma Cavalli, Brigitte von Rechenberg, Marcy Zenobi-Wong and Salim E. Darwiche in CARTILAGE

sj-tiff-6-car-10.1177_1947603521989417 – Supplemental material for Combination of a Collagen Scaffold and an Adhesive Hyaluronan-Based Hydrogel for Cartilage Regeneration: A Proof of Concept in an Ovine Model Click here for additional data file. Supplemental material, sj-tiff-6-car-10.1177_1947603521989417 for Combination of a Collagen Scaffold and an Adhesive Hyaluronan-Based Hydrogel for Cartilage Regeneration: A Proof of Concept in an Ovine Model by Clara Levinson, Emma Cavalli, Brigitte von Rechenberg, Marcy Zenobi-Wong and Salim E. Darwiche in CARTILAGE
