# Supplementary material for: Combination of a Collagen Scaffold and an Adhesive Hyaluronan-Based Hydrogel for Cartilage Regeneration: A Proof of Concept in an Ovine Model
Source: Cartilage. 2021 Jan 29;13(2 Suppl):636S–649S. doi: 10.1177/1947603521989417 (PMC8721621; doi:10.1177/1947603521989417)
Supplement: sj-docx-1-car-10.1177_1947603521989417 – Supplemental material for Combination of a Collagen Scaffold and an Adhesive Hyaluronan-Based Hydrogel for Cartilage Regeneration: A Proof of Concept in an Ovine Model [file sj-docx-1-car-10.1177_1947603521989417.docx]

**Supplementary Table 1** Modified O’Driscoll scores detailed table

**Supplementary Table 2** Modified Little scores detailed table

**Supplementary Table 3** Cyst score detailed table
