# Supplementary figures and images for: Combination of a Collagen Scaffold and an Adhesive Hyaluronan-Based Hydrogel for Cartilage Regeneration: A Proof of Concept in an Ovine Model
Source: Cartilage. 2021 Jan 29;13(2 Suppl):636S–649S. doi: 10.1177/1947603521989417 (PMC8721621; doi:10.1177/1947603521989417)

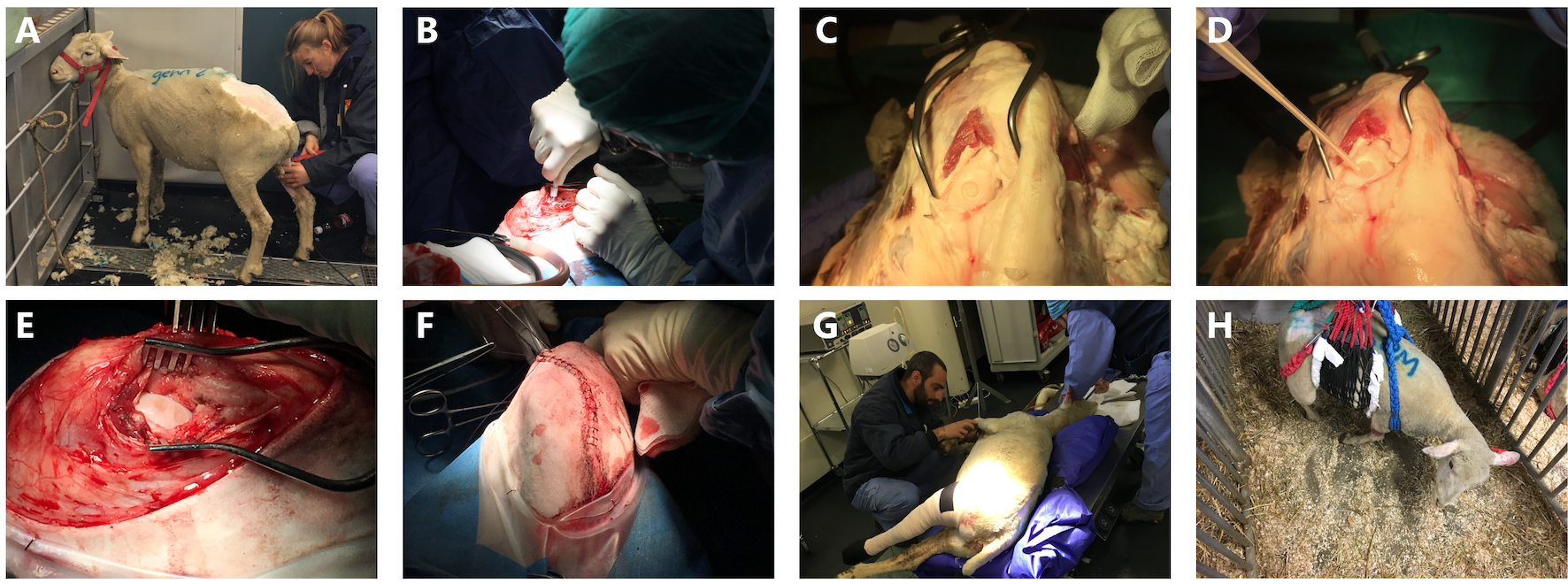

Supplement: sj-tif-2-car-10.1177_1947603521989417 – Supplemental material for Combination of a Collagen Scaffold and an Adhesive Hyaluronan-Based Hydrogel for Cartilage Regeneration: A Proof of Concept in an Ovine Model [file sj-tif-2-car-10.1177_1947603521989417.tif]

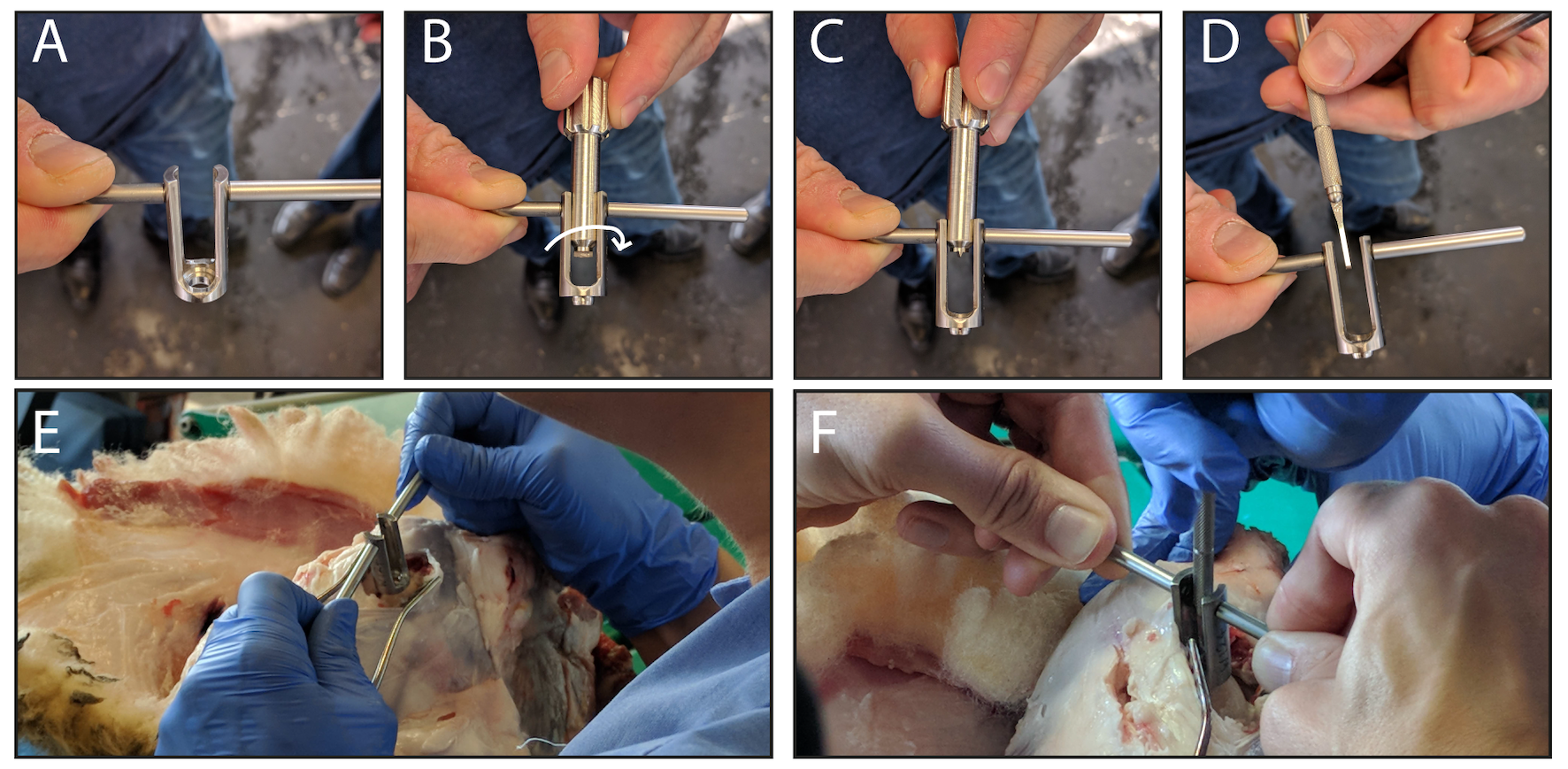

Supplement: sj-tif-3-car-10.1177_1947603521989417 – Supplemental material for Combination of a Collagen Scaffold and an Adhesive Hyaluronan-Based Hydrogel for Cartilage Regeneration: A Proof of Concept in an Ovine Model [file sj-tif-3-car-10.1177_1947603521989417.tif]

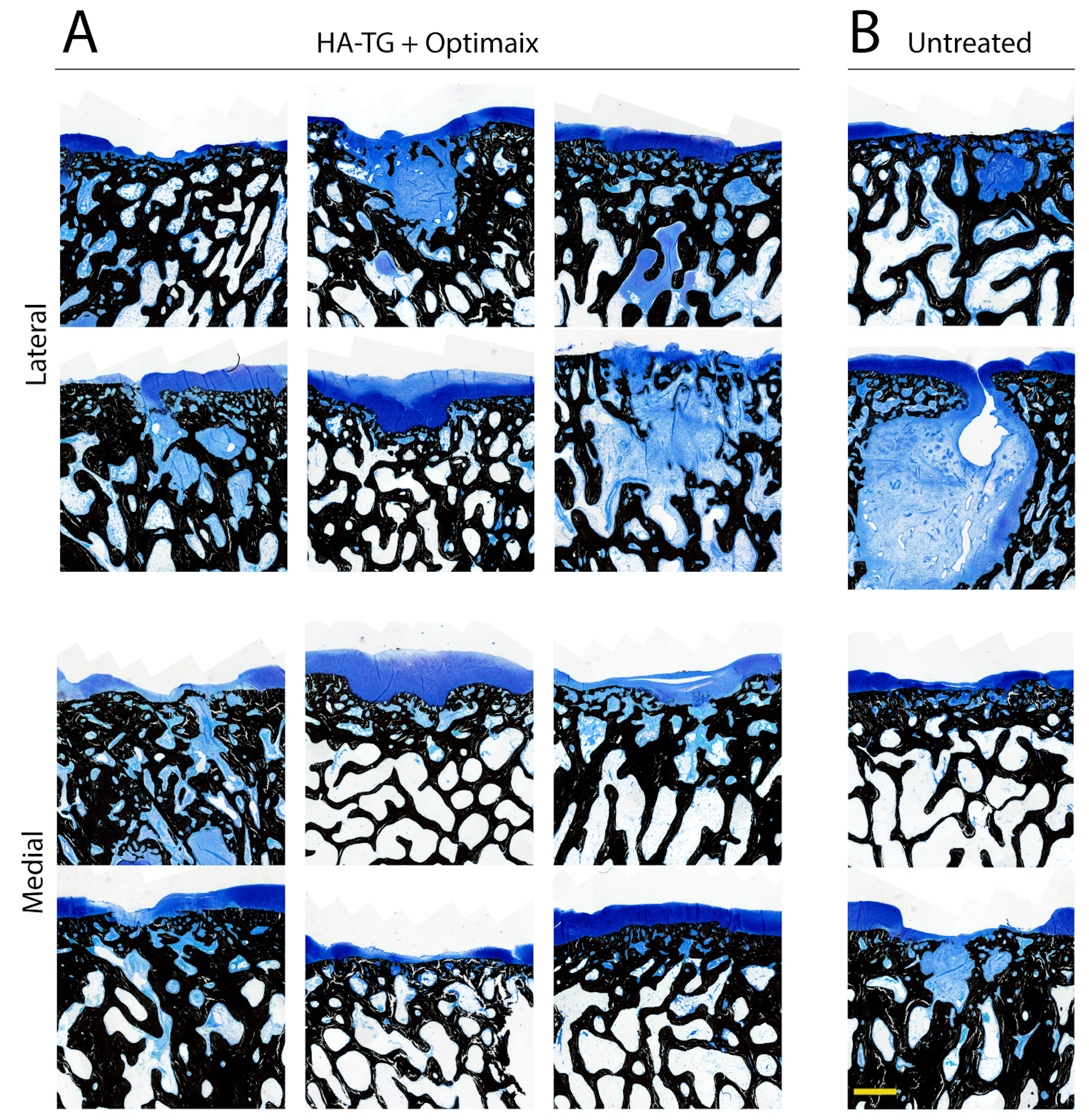

Supplement: sj-tif-4-car-10.1177_1947603521989417 – Supplemental material for Combination of a Collagen Scaffold and an Adhesive Hyaluronan-Based Hydrogel for Cartilage Regeneration: A Proof of Concept in an Ovine Model [file sj-tif-4-car-10.1177_1947603521989417.tif]

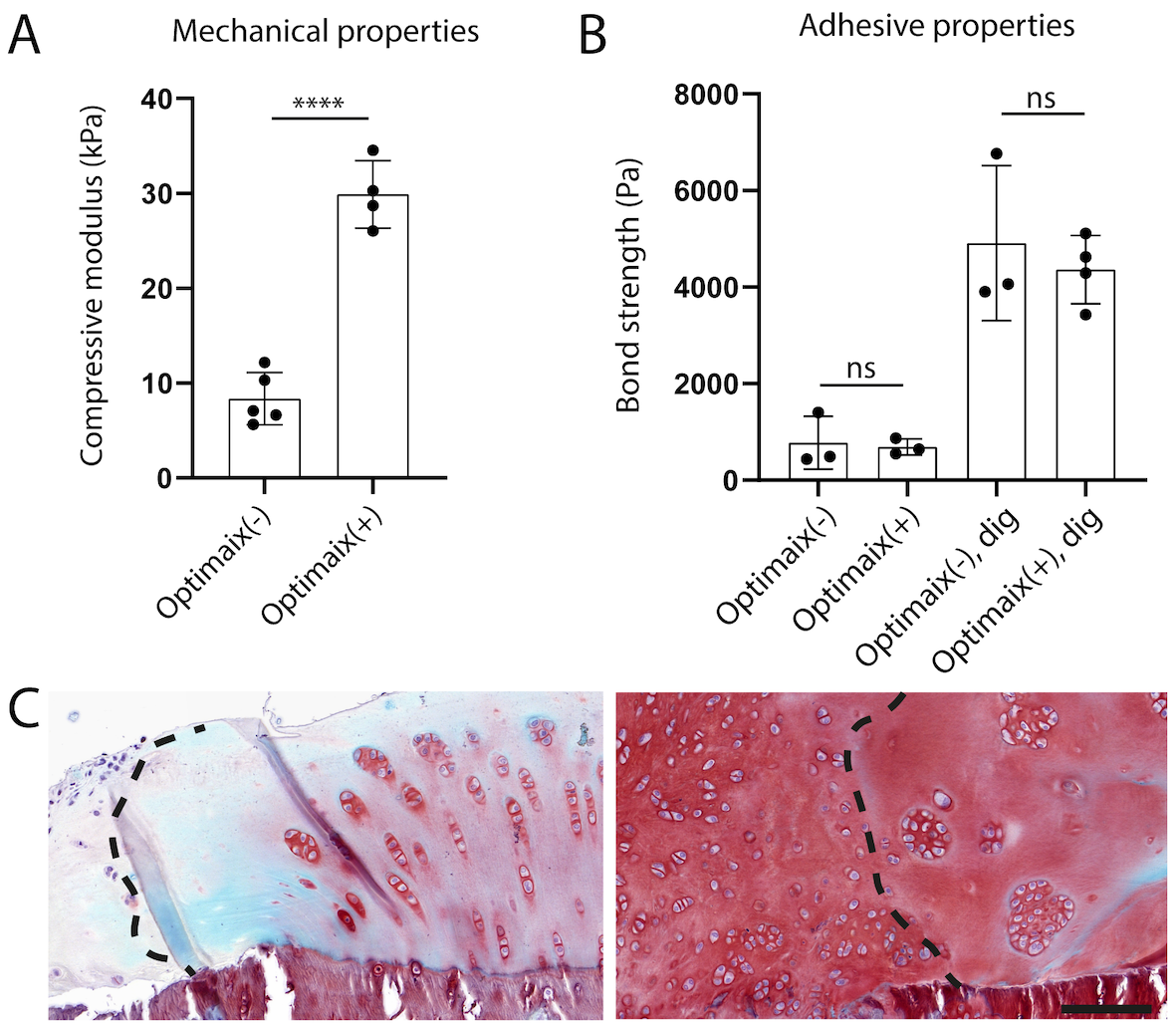

Supplement: sj-tif-5-car-10.1177_1947603521989417 – Supplemental material for Combination of a Collagen Scaffold and an Adhesive Hyaluronan-Based Hydrogel for Cartilage Regeneration: A Proof of Concept in an Ovine Model [file sj-tif-5-car-10.1177_1947603521989417.tif]

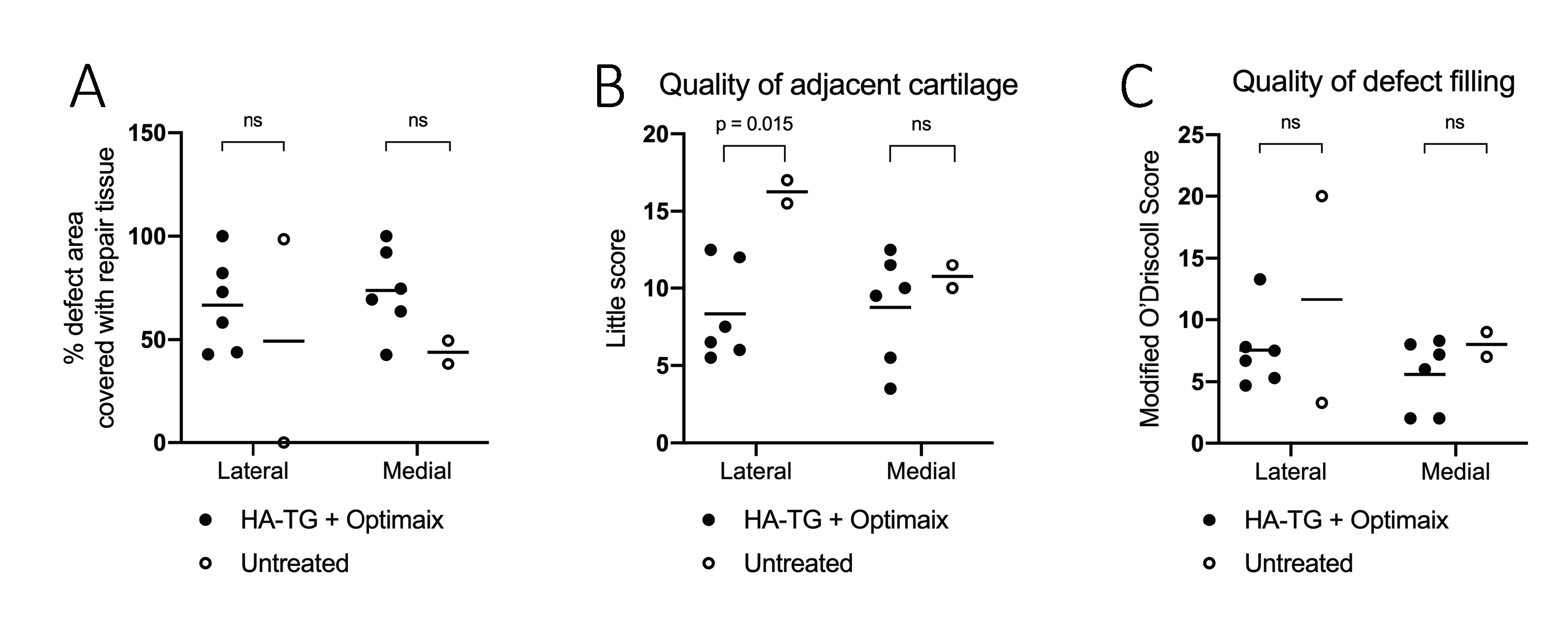

Supplement: sj-tiff-6-car-10.1177_1947603521989417 – Supplemental material for Combination of a Collagen Scaffold and an Adhesive Hyaluronan-Based Hydrogel for Cartilage Regeneration: A Proof of Concept in an Ovine Model [file sj-tiff-6-car-10.1177_1947603521989417.tiff]
